# Supplementary material for: Metabolic signatures in human follicular fluid identify lysophosphatidylcholine as a predictor of follicular development
Source: Commun Biol. 2022 Jul 29;5:763. doi: 10.1038/s42003-022-03710-4 (PMC9334733; doi:10.1038/s42003-022-03710-4)
Supplement: Supplementary file 2 — Supplementary Information [file 42003_2022_3710_MOESM2_ESM.pdf]

Supplementary Material for

"Metabolic signatures in human follicular fluid identify lysophosphatidylcholine as a predictor of follicular development"

Jihong Yang[1], Yangbai Li[1], Suying Li[1], Yan Zhang[1], Ruizhi Feng[2, 3], Rui Huang[4], Minjian Chen[4, 5*] and Yun Qian[1*]

[1] Reproductive Medical Center of Second Affiliated Hospital of Nanjing Medical University, Nanjing 210011, China.

[2] State Key Laboratory of Reproductive Medicine, Nanjing Medical University, Nanjing 211166, China.

[3] The Second Affiliated Hospital of Nanjing Medical University, Nanjing 210011, China.

[4] Key Laboratory of Modern Toxicology of Ministry of Education, School of Public Health, Nanjing Medical University, Nanjing 211166, China.

[5] State Key Laboratory of Reproductive Medicine, Center for Global Health, School of Public Health, Nanjing Medical University, Nanjing 211166, China.

* Corresponding author. These authors jointly supervised this work.

To whom correspondence: E-mail: minjianchen@njmu.edu.cn; qianyun@njmu.edu.cn

Supplementary Figures:

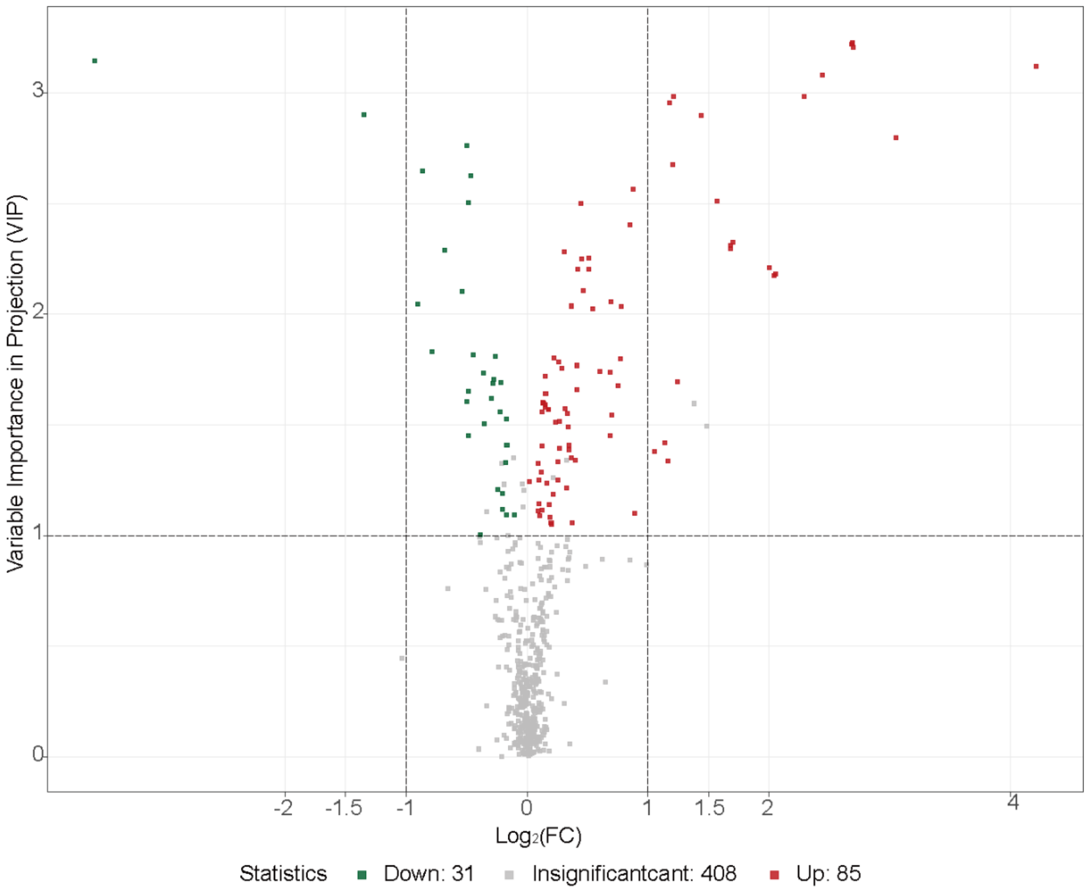

Supplementary Figure 1. Volcano plot of 116 differential metabolites. The 116 metabolites with fold change ≥2 or ≤ 0.5, VIP > 1, and $p < 0.05$ were identified.

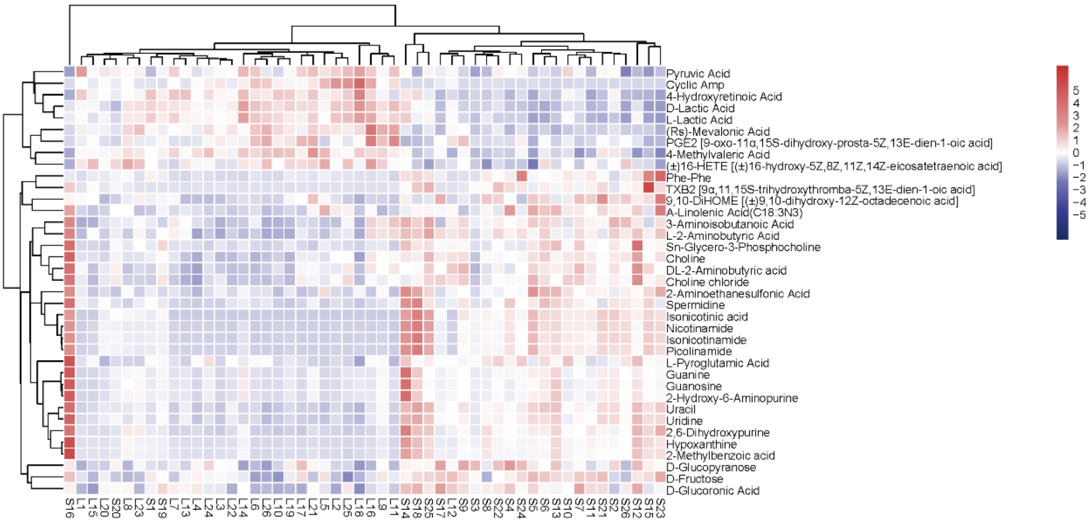

Supplementary Figure 2. Heatmap of 37 differential metabolites. Among the 116 differential metabolites, 37 metabolites with $p < 0.03$ and VIP ≥ 2 were selected for heatmap display.

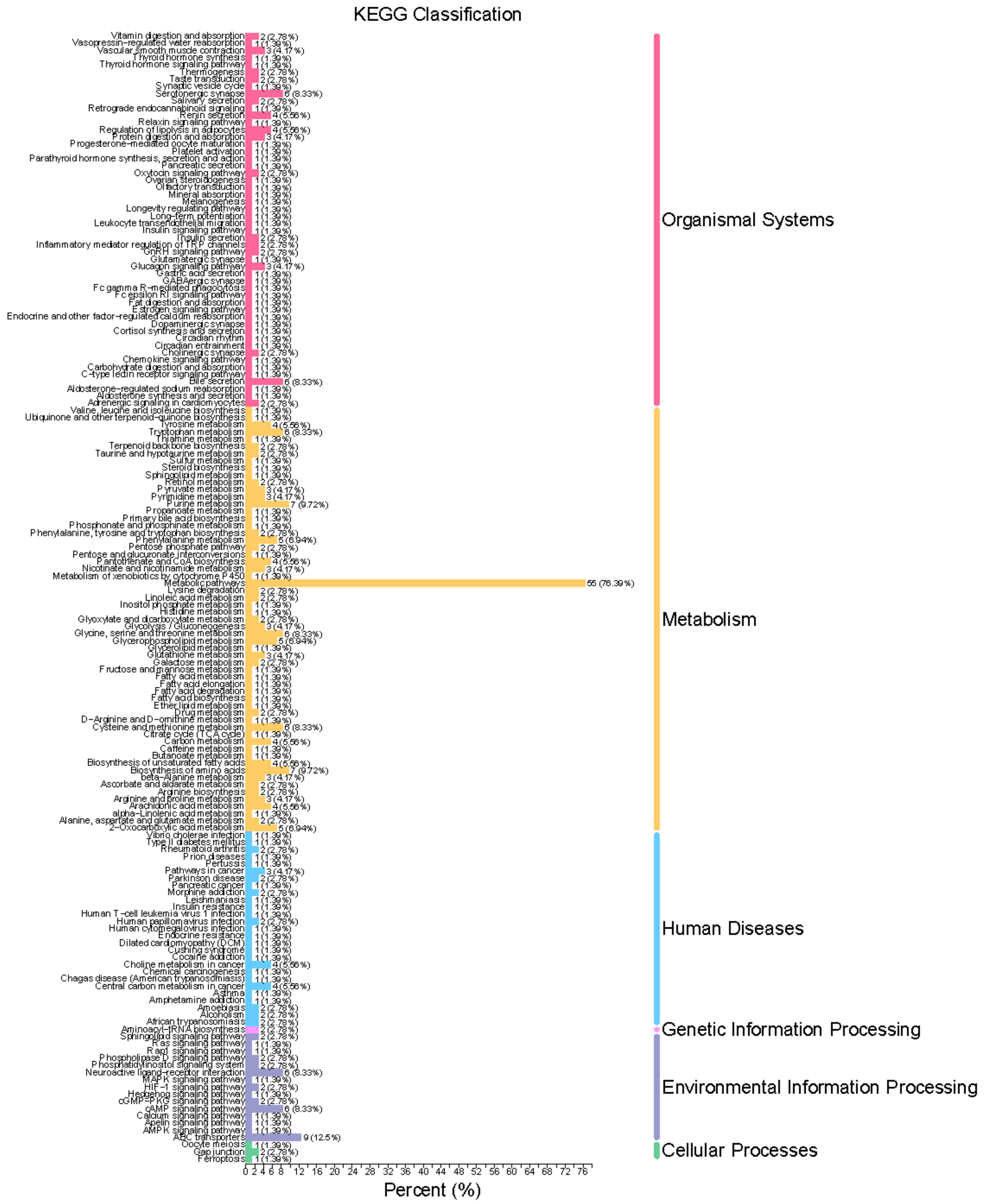

Supplementary Figure 3. KEGG classification of differential metabolites based on pathway types.

Supplementary Table 1 Clinical epidemiological information for participants who provided the FF sample (N = 26).

Characteristics	Values
Age (year)	29.50 ± 0.62
BMI	21.21 ± 0.38
Percentage of infertility type (%)	
Primary infertility	57.69
Secondary infertility	42.31
Basal E2 (pg/ml)	50.99 ± 4.48
Basal LH (mIU/ml)	4.51 ± 0.33
Basal FSH (mIU/ml)	8.24 ± 0.69
AMH (ng/ml)	4.18 ± 0.49
AFC	15.38 ± 1.01
Gn dose (IU)	2336.54 ± 107.90
Gn duration (days)	9.81 ± 0.36
E2 level on the trigger day (pmol/L)	4136.54 ± 353.29
Progesterone level on the trigger day (nmol/L)	0.98 ± 0.10
LH level on the trigger day (IU/L)	1.09 ± 0.12
No. of oocytes retrieved	13.27 ± 1.24
Oocyte maturation rate (%)	77.05 ± 3.35
Fertilization rate (%)	78.42 ± 3.02
Cleavage rate (%)	98.84 ± 2.30
High-quality embryo rate (%)	31.53 ± 4.83
Biochemical pregnancy rate (%)	65.33 ± 8.90
Clinical pregnancy rate (%)	53.33 ± 9.39

Data are presented as mean values and standard error of the mean.

For one participant, oocyte maturation rate = number of oocytes at MII (metaphase II oocytes, 1st polar body) stage/ total number of oocytes retrieved.

Fertilization rate = number of fertilized embryos/total number of oocytes retrieved.

Cleavage rate = number of cleavage embryos/number of 2PN.

High-quality embryo rate = number of high-quality embryos (grade I and II embryos)/number of 2PN.

Biochemical pregnancy rate refers to the cumulative biochemical pregnancy rate in the oocyte cycle during which the samples were collected, that is, the number of biochemical pregnancies of the participant/the number of embryos transplanted in this cycle.

Clinical pregnancy rate refers to the cumulative clinical pregnancy rate in the oocyte cycle during which the samples were collected, that is, the number of clinical pregnancies of the participant/the number of embryos transplanted in this cycle.

Supplementary Table 2 General information of participants' male spouses (N = 26).

Characteristics	Values
Age (year)	30.83 ± 0.74
BMI	24.71 ± 0.58
Semen volume (ml)	3.84 ± 0.33
Sperm density (* 10^6/ml)	144.11 ± 22.53
Sperm motility (%)	80.83 ± 1.52
Sperm deformity (%)	83.08 ± 0.68
Sperm fragmentation rate (%)	15.83 ± 2.68

Data are presented as mean values and standard error of the mean.

Supplementary Table 3 Pathway analysis of the 116 differential metabolites.

Pathway	Total	Impact	P	Metabolites
Purine metabolism	65	0.045	0.021	Xanthine; 3',5'-Cyclic AMP; Adenosine; Hypoxanthine; Inosine; Guanine; Guanosine
Pantothenate and CoA biosynthesis	19	0.043	0.047	Pantetheine; L-Aspartate; Uracil
Glycine, serine and threonine metabolism	33	0.073	0.053	Choline; N,N-Dimethylglycine; L-Cystathionine; Pyruvate
beta-Alanine metabolism	21	0	0.061	L-Aspartate; Uracil; Spermidine
Phenylalanine metabolism	10	0.595	0.068	L-Phenylalanine; Phenethylamine
Pyruvate metabolism	22	0.291	0.068	(R)-Lactate; Pyruvate; (S)-Lactate
Glycerophospholipid metabolism	36	0.098	0.069	Choline; Acetylcholine; Ethanolamine phosphate; sn-Glycero-3-phosphocholine

Note: The pathways with $p < 0.10$ were included.

Supplementary Table 4 General information of participants' male spouses (N = 200).

Characteristics	Values
Age (year)	33.78 ± 0.45
BMI	25.29 ± 0.23
Semen volume (ml)	3.40 ± 0.11
Sperm density (* 10^6/ml)	107.44 ± 5.25
Sperm motility (%)	80.02 ± 0.64
Sperm deformity (%)	83.02 ± 0.17
Sperm fragmentation rate (%)	13.64 ± 0.89

Data are presented as mean values and standard error of the mean.

Supplementary Table 5 IVF indicators and pregnancy outcomes divided by serum LPC tertiles.

**Characteristics**	**Group 1 (N = 60)**	**Group 2 (N = 74)**	**Group 3 (N = 66)**	**P**
LPC in serum (pg/ml)	<150	150-250	>250	
Number of oocytes retrieved	10.17	11.24	11.17	0.702
Fertilization rate (%)	73.89	78.64	76.35	0.476
Cleavage rate (%)	97.95	98.85	98.31	0.641
Transplantable embryo rate (%)	65.36	64.63	65.06	0.989
Biochemical pregnancy rate (%)	60.88	53.50	63.60	0.421
Clinical pregnancy rate (%)	53.74	43.72	55.12	0.301

One-way ANOVA was used to analyze differences among three groups divided by tertiles.

Supplementary Table 6 IVF indicators and pregnancy outcomes divided by FF LPC tertiles.

Characteristics	Group 1 (N = 69)	Group 2 (N = 74)	Group 3 (N = 57)	P
LPC in FF (pg/ml)	<180	180-280	>280	
Number of oocytes retrieved	10.19	11.81	10.56	0.450
Fertilization rate (%)	77.23	75.23	77.11	0.839
Cleavage rate (%)	98.17	98.73	98.40	0.842
Transplantable embryo rate (%)	66.26	65.36	63.17	0.846
Biochemical pregnancy rate (%)	59.48	56.88	57.81	0.948
Clinical pregnancy rate (%)	54.60	44.69	52.95	0.420

One-way ANOVA was used to analyze differences among three groups divided by tertiles.
