# Supplementary material for: Metabolic signatures in human follicular fluid identify lysophosphatidylcholine as a predictor of follicular development
Source: Commun Biol. 2022 Jul 29;5:763. doi: 10.1038/s42003-022-03710-4 (PMC9334733; doi:10.1038/s42003-022-03710-4)
Supplement: Supplementary file 3 — Description of Additional Supplementary Files [file 42003_2022_3710_MOESM3_ESM.pdf]

Description of Additional Supplementary Files

File name: Supplementary Data 1
Description: Metabolomic analysis based on the coverage of a wide range of targeted metabolomics.
