# Supplementary material for: Metabolic signatures in human follicular fluid identify lysophosphatidylcholine as a predictor of follicular development
Source: Commun Biol. 2022 Jul 29;5:763. doi: 10.1038/s42003-022-03710-4 (PMC9334733; doi:10.1038/s42003-022-03710-4)
Supplement: Supplementary file 5 — Reporting Summary [file 42003_2022_3710_MOESM5_ESM.pdf]

nature portfolio

Corresponding author(s): COMMSBIO-21-3507C

Last updated by author(s): Jul 14, 2022

Reporting Summary

Nature Portfolio wishes to improve the reproducibility of the work that we publish. This form provides structure for consistency and transparency in reporting. For further information on Nature Portfolio policies, see our Editorial Policies and the Editorial Policy Checklist.

Statistics

For all statistical analyses, confirm that the following items are present in the figure legend, table legend, main text, or Methods section.

n/a	Confirmed	
☐	☒	The exact sample size (n) for each experimental group/condition, given as a discrete number and unit of measurement
☐	☒	A statement on whether measurements were taken from distinct samples or whether the same sample was measured repeatedly
☐	☒	The statistical test(s) used AND whether they are one- or two-sided<br>*Only common tests should be described solely by name; describe more complex techniques in the Methods section.*
☒	☐	A description of all covariates tested
☒	☐	A description of any assumptions or corrections, such as tests of normality and adjustment for multiple comparisons
☒	☐	A full description of the statistical parameters including central tendency (e.g. means) or other basic estimates (e.g. regression coefficient) AND variation (e.g. standard deviation) or associated estimates of uncertainty (e.g. confidence intervals)
☐	☒	For null hypothesis testing, the test statistic (e.g. F, t, r) with confidence intervals, effect sizes, degrees of freedom and P value noted<br>*Give P values as exact values whenever suitable.*
☒	☐	For Bayesian analysis, information on the choice of priors and Markov chain Monte Carlo settings
☒	☐	For hierarchical and complex designs, identification of the appropriate level for tests and full reporting of outcomes
☐	☒	Estimates of effect sizes (e.g. Cohen's d, Pearson's r), indicating how they were calculated

Our web collection on statistics for biologists contains articles on many of the points above.

Software and code

Policy information about availability of computer code

Data collection	No software was uesd.
Data analysis	No software was uesd.

For manuscripts utilizing custom algorithms or software that are central to the research but not yet described in published literature, software must be made available to editors and reviewers. We strongly encourage code deposition in a community repository (e.g. GitHub). See the Nature Portfolio guidelines for submitting code & software for further information.

Data

Policy information about availability of data

All manuscripts must include a data availability statement. This statement should provide the following information, where applicable:

- Accession codes, unique identifiers, or web links for publicly available datasets
- A description of any restrictions on data availability
- For clinical datasets or third party data, please ensure that the statement adheres to our policy

All data generated or analysed during this study are included in this published article (and its supplementary information files).

Human research participants

Policy information about studies involving human research participants and Sex and Gender in Research.

Reporting on sex and gender	The population samples included in this study were all From Asian women.
Population characteristics	Infertility in these women were brought on by either tubal complication or unknown cause of sterility. Unknown cause of sterility, in this research, was described as infertility even after two attempts of intrauterine insemination. These women had normal follicular development and none of the offered interventions affected follicular development, thus reducing errors, relative to other populations. We also selected women whose male partners were willing and able to provide healthy sperm for IVF. This was put in place to reduce effects of male compromising factors on the results. The patients excluded from the study were: patients with a history of endometriosis, polycystic ovary syndrome, premature ovarian failure, ovarian surgery, or other factors.
Recruitment	We recruited women who underwent in vitro fertilization during their initial cycle of ovarian stimulation, as part of an agonist protocol. All participants were recruited at the Reproductive Center of the Second Affiliated Hospital of Nanjing Medical University, China, and provided written informed consent.
Ethics oversight	We received ethical approval from the Second Affiliated Hospital of Nanjing Medical University.

Note that full information on the approval of the study protocol must also be provided in the manuscript.

Field-specific reporting

Please select the one below that is the best fit for your research. If you are not sure, read the appropriate sections before making your selection.

☒ Life sciences ☐ Behavioural & social sciences ☐ Ecological, evolutionary & environmental sciences

For a reference copy of the document with all sections, see nature.com/documents/nr-reporting-summary-flat.pdf

Life sciences study design

All studies must disclose on these points even when the disclosure is negative.

Sample size	A total of 452 samples based on a two-stage study design.
Data exclusions	No data were excluded from the analyses.
Replication	All attempts at replication were successful.
Randomization	Allocation was not random. In this study, a total of 226 infertile women, with tubal complications or unexplained sterility, experiencing their first in vitro fertilization cycle.
Blinding	We classified and analyzed according to the basic situation.

Reporting for specific materials, systems and methods

We require information from authors about some types of materials, experimental systems and methods used in many studies. Here, indicate whether each material, system or method listed is relevant to your study. If you are not sure if a list item applies to your research, read the appropriate section before selecting a response.

Materials & experimental systems

n/a	Involved in the study
☒	☐ Antibodies
☒	☐ Eukaryotic cell lines
☒	☐ Palaeontology and archaeology
☒	☐ Animals and other organisms
☒	☐ Clinical data
☒	☐ Dual use research of concern

Methods

n/a	Involved in the study
☒	☐ ChIP-seq
☒	☐ Flow cytometry
☒	☐ MRI-based neuroimaging
